# FAMILY CAREGIVING FOR INDIVIDUALS WITH PARKINSON’S DISEASE AND LEWY BODY DEMENTIA IN CALIFORNIA

**DOI:** 10.1093/geroni/igae098.0775

**Published:** 2024-12-31

**Authors:** Robin Whitney, Janice Bell, Benjamin Link, Shikha Bhurtel, Everlyne Ogugu, Orly Tonkikh, Kathleen Kelly, Heather Young

**Affiliations:** San Jose State, San Jose, California, United States; UC Davis, Davis, California, United States; University of California Davis, Davis, California, United States; UC Davis, Davis, California, United States; University of California Davis, Davis, California, United States; University of California Davis, Davis, California, United States; Family Caregiving Alliance, San Francisco, California, United States; UC Davis, Ashland, Oregon, United States

## Abstract

Parkinson’s Disease (PD) and Lewy Body Dementia (LBD) are related progressive neurodegenerative disorders that primarily affect older adults. Many individuals with PD/LBD require support from a family caregiver. However, caregivers of individuals with PD/LBD are infrequently studied. We extracted data from 2019-2022 in CareNavTM, a statewide electronic platform with data from caregivers served by the eleven California Caregiver Resource Centers, to compare caregiver sociodemographic characteristics, caregiving activities, and outcomes among caregivers of individuals with PD/LBD to those of individuals with Alzheimer’s Disease and related dementia (ADRD) and other chronic conditions (OCC). Care recipient condition was examined as a predictor of caregiving outcomes (e.g., loneliness, depressive symptoms, strain, and worsening health) using multivariable logistic regression models. There were a total of n=13,649 caregivers, with a majority caring for someone with ADRD (66%), followed by OCC (23%), and PD/LBD (10%). PD/LBD caregivers were more likely to be older, female, white, college-educated, and retired. PD/LBD caregivers were more likely to care for a spouse, spend >40hrs/wk on caregiving, assist with more personal care, and experience problems with the care recipient waking others up at night. In multivariable models, PD/LBD caregiving was independently associated with caregiving strain (OR 1.2; 95% CI [1.0-1.4], p= 0.01) and worsening health (OR 1.2; 95% I [1.1-1.4], p< 0.01), but not with depressive symptoms or loneliness. PD/LBD caregivers are a unique subset of family caregivers engaged in intense and complex care. Services and supports tailored to this condition and related needs may be warranted to optimize caregiver wellbeing.

